# Effects of two-dose ceftiofur treatment for metritis on the temporal dynamics of antimicrobial resistance among fecal *Escherichia coli* in Holstein-Friesian dairy cows

**DOI:** 10.1371/journal.pone.0220068

**Published:** 2019-07-22

**Authors:** Ethan A. Taylor, Ellen R. Jordan, Jose A. Garcia, Gerrit R. Hagevoort, Keri N. Norman, Sara D. Lawhon, Juan M. Piñeiro, Harvey M. Scott

**Affiliations:** 1 Department of Veterinary Pathobiology, Texas A&M University, College Station, TX, United States of America; 2 Department of Animal Science, Texas A&M University, Dallas, TX, United States of America; 3 Department of Extension Animal Sciences and Natural Resources, New Mexico State University, Clovis, NM, United States of America; 4 Department of Veterinary Integrative Biosciences, Texas A&M University, College Station, TX, United States of America; 5 Department of Animal Science, Texas A&M University, Amarillo, TX, United States of America; Tokat Gaziosmanpasa University, TURKEY

## Abstract

A pair-matched longitudinal study conducted on three dairy farms in the U.S. High-Plains explored the temporal effects of two-dose ceftiofur crystalline-free acid (CCFA) treatment for metritis on third-generation cephalosporin (3GC) resistance among enteric *E*. *coli* in Holstein-Friesian cows. The current 13-day slaughter withholding period does not account for rising populations of third-generation cephalosporin (3GC) resistant bacteria in feces of animals following CCFA treatment. A total of 124 matched-pairs of cows were enrolled in the study. Cows diagnosed with postpartum metritis received the product twice at the labeled dose of 6.6 mg/kg subcutaneously at the base of alternating ears. Untreated cows–absent clinical metritis–were matched on lactation number and calving date. Feces were collected *per rectum* on days 0 (baseline), 6, 16, 28, and 56. Environmental samples, from watering troughs as well as surface manure from fresh-cow, hospital, maternity, and milking pens, and from the compost pile were collected prior to the animal sample collection period. Historical data on metritis rates and CCFA use were compiled from herd records. On day 0, cows exhibited an overall mean difference of over 4 log_10_ colony forming units (CFU) comparing 3GC resistant *E*. *coli* to the general *E*. *coli* population. At the first eligible slaughter date, the difference declined to 3.31 log_10_ CFU among cows in the CCFA group (P<0.01 compared to control cows). Such differences were no longer observed between the treated and control groups by day 28. Results suggest a 13-day withholding period following the final treatment is insufficient to allow levels of 3GC resistant *E*. *coli* to return to baseline. This effect varied by farm and was dependent upon the starting level of resistance. A farm-specific extended slaughter-withholding period could reduce the microbial risk to food products at slaughter.

## Introduction

Over the past five decades, antimicrobial resistance (AMR) among bacteria of clinical importance has continued to expand its threat to public health, drawing the attention of national and international agencies, researchers, and industry [1]. Among the most threatening are bacteria with resistance to third (and higher) generation cephalosporins. The World Health Organization considers these antibiotic classes to be critically important and of highest priority to human medicine [2]. Of the antibiotics within these classes, ceftiofur, a third-generation cephalosporin (3GC), is the only drug labeled for use in U.S. animal agriculture; however, it is available in several formulations reflecting differences in ease-of-use, dosage and route of administration, and duration of activity.

Metritis is a common postpartum disease in dairy cows, clinically expressed as fever above 39.5°C, with a thickened uterine wall, and emitting a malodorous reddish-brown discharge [3]. These symptoms typically occur within 21 days after calving [3]. Metritis imposes an economic burden on production, as an estimated $4.70 is spent per head of cattle in each herd per year to prevent and treat metritis [4]. Additionally, metritis decreases milk production [5, 6] and is associated with an increased time between calving and conception, thus impeding herd productivity and profitability in several ways [7]. Prior to April 2012, a single-dose treatment with ceftiofur crystalline-free acid (CCFA) was on-label for treatment of metritis; subsequently, a two-dose treatment is now required to remain on-label. Antibiotics, such as CCFA, are frequently used to treat metritis in dairy cows; however, penicillin, ampicillin, and oxytetracycline also can be prescribed in dairy production settings [8–11]. Arguably, a two-dose CCFA treatment is required due to drug concentrations in uterine tissues decreasing too quickly in a single dose regimen [12]. When an initial shorter-term formulation of ceftiofur was approved in 1988 [13], extralabel cephalosporin administration was not prohibited, though prescriptions by a veterinarian were required [14]. This has since changed, as the U.S. Food and Drug Administration (FDA) banned most extra-label uses of cephalosporins in food animals in 2012 [15].

CCFA is commonly used in dairy herds due to a zero-day milk withholding period [14, 16]. Nevertheless, section 522.313a of the U.S. Code of Federal Regulations Title 21, Chapter 1, requires a 13-day meat withholding period from the last date of administration of CCFA [17]. The risks posed by food products contaminated with commensal and pathogenic enteric bacteria have been widely discussed [18–23] and much research has been performed showing CCFA usage in dairy production medicine leads to increased levels of 3GC resistance among *E*. *coli*, most often in the form of increased cephamycinase (e.g., *bla*_CMY-2_) and extended-spectrum beta-lactamase (ESBL; e.g., *bla*_CTX-M-32_) gene presence [13, 14, 24–26]. However, a literature gap remains concerning how the 2012 two-dose CCFA regimen for the treatment of metritis in dairy cattle directly impacts levels of antimicrobial resistance among enteric bacteria, such as *E*. *coli* populations. McEwen [27] noted a generalized worldwide focus on antibiotic residues in retail meats and a lack of policy aimed at reducing the risk of antibiotic resistant bacteria coming through the food chain.

The present study is inspired by a risk assessment framework divided into release, exposure, and consequence assessments [28, 29]. It details factors involved in resistant bacteria or their determinants “escaping” the farm and presenting exposure to humans through retail meats contaminated with resistant bacteria at slaughter via fecal contamination [28, 29]. Importantly, meat quality is not of concern, nor is it under consideration in this study. Contamination of beef products with fecal bacteria has previously been observed [20] and linked to disease in human populations both with [30] and without AMR being considered [21–23, 31]. Our study aim was to evaluate how two-dose treatment with CCFA impacts enteric bacterial populations, whether or not the labeled slaughter withholding period is sufficient to allow 3GC resistance among bacterial populations to return to pre-treatment levels, and how much farm-to-farm variability, including prior treatment history and pre-existing levels of 3GC resistance, might affect these dynamics. Furthering this understanding will aid regulators and stakeholders in developing antimicrobial stewardship principles aimed at reducing the risk to public health from cephalosporin-resistant enteric bacteria.

## Materials and methods

### Study design

A pair-matched longitudinal study was employed to evaluate the effects of two-dose administration of CCFA for the treatment of metritis on the temporal dynamics of 3GC resistance among enteric *E*. *coli* in Holstein-Friesian dairy cattle. Three large U.S. southwestern dairy farms, representative of the style of farming in the region, were purposively identified and enrolled in the study. All producers provided consent to perform the study on their farm and were provided both the antibiotic product CCFA (Excede, Zoetis Animal Health, Florham Park, NJ) at no cost and financial compensation for use of their cattle. The animal experiments were approved by the Texas A&M University Institutional Animal Care and Use Committee (Protocol No. 2016–0183). Farms were located in either northwestern Texas (n = 2) or eastern New Mexico (n = 1). Herds ranged from 3,400–5,400 head of cattle with housing styles common to the region. Herds were enrolled into the study based on their large size, which was necessary to provide the expected number of metritis cases in a one year period, and on previous working relations with the extension services of Texas A&M University System or New Mexico State University. Cattle with postpartum metritis were diagnosed either by the licensed herd veterinarian, or using the herd veterinarian’s established protocol, and prescribed a two-dose regimen of CCFA. Diagnosis criteria were the same for both licensed veterinarian and herd-veterinarian established protocols including fever at or above 39.5°C, a malodorous reddish-brown discharge, and a thickened uterine wall. During the first season (spring/summer), 46 pairs of cattle (15 on each of Dairy Farms 1 and 3, and 16 on Dairy Farm 2) were enrolled with sample collection occurring from April to July of 2017. The second season (autumn/winter) had 78 pairs of cattle (27 on Dairy Farm 1, 26 on Dairy Farm 2, and 25 on Dairy Farm 3) enrolled with sample collection from September of 2017 to April of 2018.

### Treatment administration

**Fig 1:**
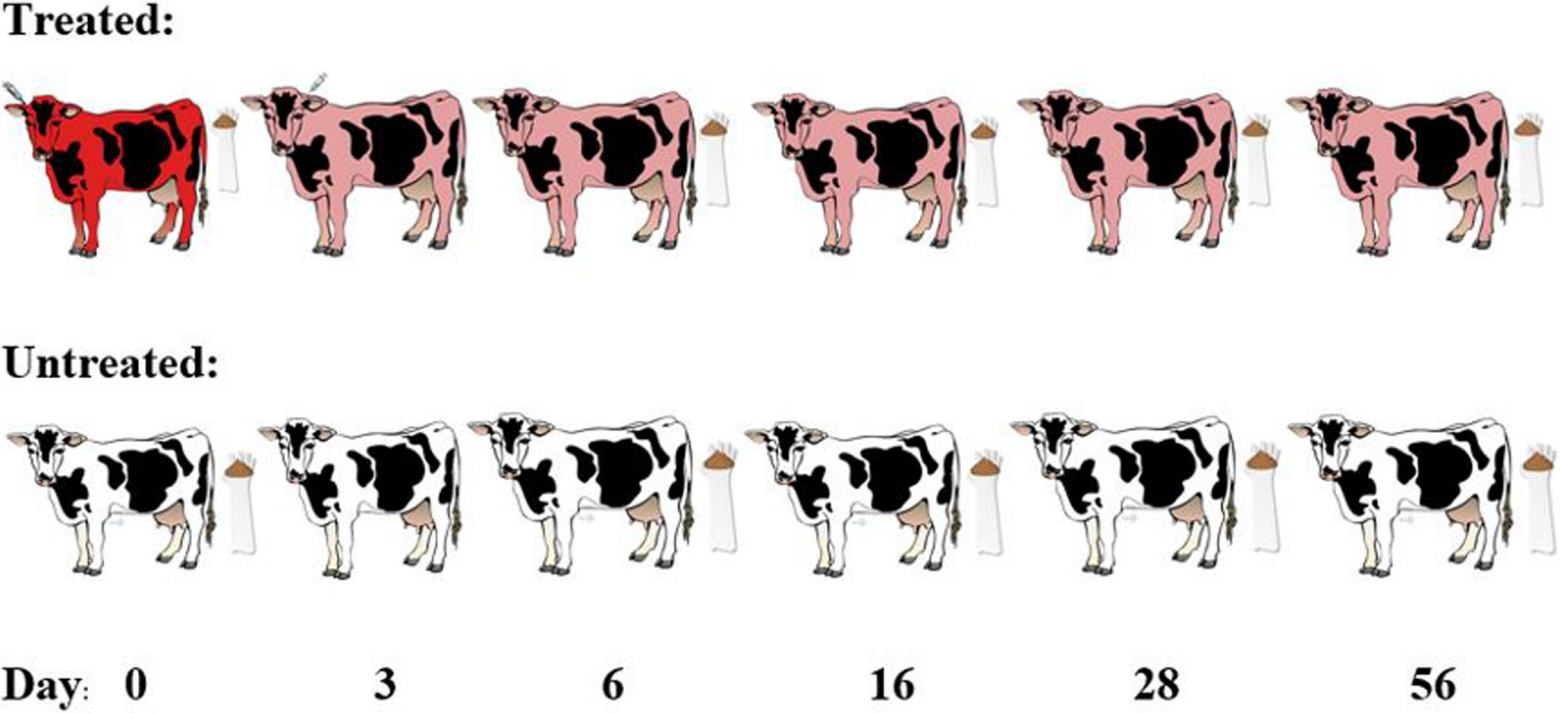
Schematic of study design. Study design is displayed above. The red cow is indicates status at metritis diagnosis, pink cows represent a cow with metritis having been treated with ceftiofur crystalline-free acid (CCFA), while white cows represent healthy pair-matched controls. The syringe by the ear of the first 2 cows in the treated group represent the administration of CCFA in contralateral ears. Gloves with feces represent days at which samples were collected. Day 0 samples were collected prior to CCFA administration.

The first dose was administered subcutaneously at the base of one ear while the second dose was given 72 hours later in the opposite ear (Fig 1). Treatment dosage was administered per label instructions at 6.6 mg/kg body weight. Upon diagnosis, cows with metritis were pair-matched by lactation number and calving date with a control cow on the same farm. Additional recorded metrics including cow age, time lag from calving date to study enrollment, other ancillary treatments, and the season of enrolment were retrieved from the computerized records system DairyComp 305 (Valley Agricultural Software, Tulare, CA). Background CCFA usage data from the previous year were obtained from historical DairyComp 305 dairy farm records. Treatment information was aggregated at the farm level as entered by dairy farm personnel. Treatments with any formulation of ceftiofur and for any clinical indication were included in determining historical usage. To calculate the ratio of animal lactations treated, the number of ceftiofur doses administered to any animal was divided by the number of cows and heifers that freshened that year. It was not possible to calculate defined daily doses (DDD) since dosage information outside the experimental study was unavailable.

### Environmental sample collection

Prior to the start of each animal study, environmental samples were collected from various locations at each dairy farm. Water samples were collected using sterile, individually packaged plastic tablespoons. Samples were collected in 2 mL aliquots from 10 locations across each dairy and placed into a single sterile 50 mL conical tube (Falcon, Corning, NY) and then diluted with sterile 50 percent glycerol at a 1:1 ratio by volume (Fisher Chemical, Thermo Fisher Scientific, Waltham, MA). An equivalent non-glycerol diluted water sample was collected similarly, but with 4 mL aliquots added from each location. In addition, 60-gram samples of manure were collected via a shoulder-length obstetric (OB) sleeve from fresh-cow, hospital, maternity, and milking pens, and from the compost pile in a diagonal transect. Twenty grams of sample was dispensed into a 50 mL conical tube containing 20 mL of sterile 50 percent glycerol solution for sample preservation. An additional 40 grams of sample were placed in an empty conical tube. Samples were stored at -80°C in the laboratory at Texas A&M University until the time at which they were analyzed.

### Animal sample collection

After being diagnosed with metritis, a baseline fecal sample was collected from the affected cow and her pair-matched control *per rectum* with an OB sleeve prior to the first CCFA treatment (Fig 1). Each treated cow with metritis was matched with a healthy cow (untreated) on the same farm having the same lactation number and recent calving date. These day 0 samples represented the pretreatment baseline for the pair. After baseline samples were collected, the first CCFA dose was given to the cow with clinical metritis and the second dose was administered 72 hours later. Subsequently, fecal samples were collected on study days 6, 16, 28, and 56. Since the second dose was administered 72 hours (3 days) after the first and the first eligible slaughter date is 13 days from the last CCFA dose, the day 16 sample represents the first-eligible slaughter day. Samples were stored in a -80°C freezer in the laboratory at Texas A&M University in College Station, TX before microbiological processing at a later time.

### Sample processing

To process the fecal and environmental samples, one milliliter of water or one gram of feces was removed from the glycerol tube and placed into a 15 mL conical tube (Falcon, Corning, NY) with 9 mL of 1x phosphate buffered saline (PBS) (Gibco, Thermo Fisher Scientific, Gaithersburg, MD) creating a 1:20 dilution (accounting for glycerol). The tube was then vortexed for approximately 10 seconds to homogenize the mixture. One mL was pipetted from the PBS mixture into a spiral-plating cup. Subsequently, a 50 μl aliquot of each sample from the PBS mixture was spiral-plated onto each of plain MacConkey agar (MAC) (BD Difco, Franklin Lakes, NJ) and MacConkey agar containing ceftriaxone (MACCEF) (Sigma-Aldrich, St. Louis, MO) at the Clinical and Laboratory Standards Institute (CLSI) clinical breakpoint minimum inhibitory concentration (MIC) of 4 μg/mL [32] using the Eddy Jet 2 instrument (Neutec Group Inc., Farmingdale, NY). MAC plates grow both 3GC-resistant and susceptible *E*. *coli* populations, while MACCEF plates only grow 3GC-resistant isolates. This allowed for quantitative comparison of the two populations across our sampling periods and between treatment groups. After spiral plating, plates were incubated for 18 hours at 37°C.

Once the incubation period was complete, plates were quantified for *E*. *coli* by counting phenotypically characteristic pink, lactose fermenting colonies using the Flash & Go instrument (Neutec Group Inc., Farmingdale, NY). One limitation of this methodology is bacteria need to grow to a population at or above 2.5 log_10_ CFU. Any viable bacterial counts below this level is give the appearance the sample is negative. After counting MAC and MACCEF plates, two colonies from each plate suspected to be *E*. *coli* were selected for isolation on tryptic soy agar with 5% sheep blood (hereafter blood agar plates; Becton, Dickinson, Franklin Lakes, NJ). Blood agar plates were incubated for 18 hours at 37°C. Isolates were then tested for their ability to convert tryptophan to indole using James Reagent (bioMérieux, Marcy-l’Étoile, France) to biochemically test for *E*. *coli*. *I*solates were further confirmed via Matrix-Assisted Laser Desorption Ionization–Time of Flight Mass Spectrometry (MALDI–TOF MS) (Bruker, Billerica, MA) to be *E*. *coli*.

### Statistical analysis

Descriptive tabulations were performed using Stata version 15.1 (StataCorp LLC, College Station, TX) to summarize the overall counts and distribution of resistance across dairy farms, as well as by treatment and sample day. Mixed effect linear regression was utilized for multivariable analysis of colony counts and differences among agar types. Mixed effect linear regression involving 3GC resistant *E*. *coli* counts was performed after plates with growth below the limit of quantification (LOQ, 2.5 log_10_ CFU) were imputed across values ranging between zero and the LOQ employing multiple imputation techniques previously described in Kanwar et al. [33]. By setting the lower limit at zero and the upper limit at 2.5 (log_10_ CFU), values initially displaying no growth were distributed across a log_10_ scale in combination with values above the LOQ to more closely reflect the CFU distribution expected in nature, thus allowing linear regression models to be utilized. Analyses of colony counts from cow samples were performed with season, dairy farm, and animal included as random effects for overall analyses. Covariance/correlation structures that were explored for repeated samples within cow included: independent, exchangeable, M-1 dependent and AR-1 autoregressive. Treatment, sample day, and historical ceftiofur usage were considered as potential fixed effects in models, along with the interaction of treatment and sampling day to account for the metabolism and excretion of the antibiotic in those models. Dairy farm was removed as a random effect and farm was considered as a fixed effect (main and interaction terms), along with the previously mentioned variables, for farm-to-farm comparisons. A P-value of 0.05 and below was considered to be statistically significant.

## Results

### Descriptive statistics

In total, 9 pen-floor samples were collected from each of fresh cow, hospital, maternity, and milking pens, along with the compost area from each dairy farm. Samples were analyzed independently and CFU means were calculated from the growth data of each sample; that is, environmental samples were not pooled. Total *E*. *coli* CFU counts were similar across all three dairy farms; however, Dairy Farm 3 had mean quantifiable levels of 3GC resistant *E*. *coli* approximately 1.5 log_10_ CFU greater than the other two farms (Dairy Farm 1 95% CI: 0.575–3.116; Dairy Farm 2 95% CI: 0.286–2.939; Dairy Farm 3 95% CI: 1.765–4.575; Table 1). This led to differences between total and 3GC growth of approximately one log_10_ CFU less than the other two dairy farms (Table 1). All water sources sampled across the three dairy farms tested negative for detectable levels of *E*. *coli*. Data can be found in the supplemental materials (S1 and S2 Datasets).

**Table 1:** Descriptive data concerning the distribution of environmental *E*. *coli* growth across three dairy farms.

	Growth Metric											
	Log_10_ Total *E*. *coli* (MAC)				Log_10_ 3GC Resistant *E*. *coli* (MACCEF)				Log_10_ Growth Arithmetic Difference (Total– 3GC)			
Dairy Farm	Mean	Standard Error	95% CI	Median	Mean	Standard Error	95% CI	Median	Mean	Standard Error	95% CI	Median
Dairy Farm 1 (n = 9)	5.241	0.183	4.864–5.618	5.220	1.846	0.618	0.575–3.116	2.603	3.396	0.637	2.085–4.706	3.593
Dairy Farm 2 (n = 9)	4.917	0.315	4.270–5.563	5.328	1.613	0.645	0.286–2.939	0	3.304	0.561	2.152–4.456	3.080
Dairy Farm 3 (n = 9)	5.564	0.259	5.032–6.097	5.858	3.170	0.683	1.765–4.575	3.556	2.395	0.550	1.263–3.526	2.620

MAC, plain MacConkey agar; MACCEF, MacConkey agar with 4 μg/mL; 3GC, third-generation cephalosporin

The mean cow lactation number was 1.7 and cows were, on average, 9.8 days in milk at the time of enrollment. The average age of cows enrolled in the study was 34.3 months and 60% of enrolled cows were primiparous. The estimated weight of first lactation animals was 545 kg, 615 kg for second lactation, and 665 kg for third and higher lactation animals on the dairy farms. Based upon the age and lactation of enrolled animals, along with the estimated weights of each lactation number on the farms, it is estimated the average weight of animals enrolled in the study was 601 kg. The treatment ratio of cows and heifers freshened in the prior year with any ceftiofur formulation was 9.0% on Dairy Farm 1, 8.4% on Dairy Farm 2, and 121.0% on Dairy Farm 3. Of those ratios, 0% from Dairy Farm 1, 23.6% from Dairy Farm 2, and 8.6% from Dairy Farm 3 were intra-mammary formulations and not systemic therapy given via injection. This means animals on Dairy Farm 1 averaged 0.090 treatments per cow-lactation, 0.084 treatments on Dairy Farm 2, and 1.210 treatments per fresh cow or heifer on Dairy Farm 3. Since ceftiofur formulations have been prohibited for off-label usage in the United States since April 2012, we assumed that each dosage of CCFA administered was at 6.6 mg per kg of body weight and the other ceftiofur formulations also were at labeled dose.

Summary statistics (mean, standard error, 95% confidence intervals and the median) by treatment group, day, dairy, and season are presented in Table 2. Treated animals had lower mean and median values of growth on MAC, but higher values of growth on MACCEF; meanwhile, the arithmetic difference between those two outcomes decreased substantially among treated animals in comparison to the untreated group (Table 2). Similar trends were observed regarding these metrics across sample day relating to the time from drug administration. There was a mean decrease in the total *E*. *coli* population on the first sampling day following treatment with an increase in mean 3GC resistant *E*. *coli* population on the same day. Thereafter, the total *E*. *coli* population increased and 3GC resistant *E*. *coli* population decreased as time progressed from treatment administration. Descriptive statistics illustrating these phenomena are shown in Table 3. Samples from Dairy Farm 3 exhibited a higher mean and median *E*. *coli* growth on both MAC and MACCEF agars, with a smaller arithmetic difference between the two when compared to Dairy Farms 1 and 2 (Table 2). Values remained steady across plate type and arithmetic difference with regards to the metrics of mean, median, and standard error for the factor of season. Sample number varies across these metrics, as some animals were culled prior to study completion or else samples were missed during the collection period (Table 2).

**Table 2:** Descriptive data on the distribution of *E*. *coli* growth within treatment, sampling day, dairy farm and season. Univariate summary statistics are unadjusted for clustering by farm, pen, or animal.

Growth Metric	Descriptive Statistic	Treatment		Sample Day					Dairy Farm			Season	
		Treated (n = 595)	Untreated (n = 596)	0 (n = 246)	6 (n = 239)	16 (n = 239)	28 (n = 237)	56 (n = 230)	1 (n = 408)	2 (n = 388)	3 (n = 395)	Spring/ Summer (n = 440)	Fall/Winter (n = 751)
Log_10_ Total *E*. *coli* (MAC)	Mean	4.343	4.752	4.641	3.927	4.584	4.759	4.838	4.245	4.445	4.963	4.686	4.467
	Standard Error	0.068	0.047	0.079	0.126	0.090	0.078	0.075	0.082	0.072	0.057	0.055	0.058
	95% CI	4.209–4.477	4.660–4.845	4.486–4.796	3.680–4.173	4.408–4.761	4.606–4.912	4.690–4.986	4.084–4.405	4.304–4.585	4.850–5.075	4.578–4.793	4.353–4.581
	Median	4.623	4.798	4.690	4.435	4.784	4.781	5.010	4.510	4.589	5.182	4.729	4.722
Log_10_ 3GC Resistant *E*. *coli* (MACCEF)	Mean	0.884	0.446	0.522	1.071	0.772	0.475	0.478	0.426	0.281	1.288	0.707	0.640
	Standard Error	0.067	0.048	0.078	0.117	0.102	0.078	0.082	0.057	0.052	0.0915	0.069	0.053
	95% CI	0.751–1.016	0.352–0.540	0.370–0.675	0.843–1.301	0.573–0.972	0.322–0.697	0.318–0.638	0.315–0.538	0.179–0.383	1.109–1.470	0.571–0.843	0.537–0.743
	75^th^ Percentile	0	0	0	2.603	0	0	0	0	0	2.904	0	0
Log_10_ Growth Arithmetic Difference (Total– 3GC)	Mean	3.459	4.306	4.119	2.855	3.812	4.285	4.360	3.819	4.164	3.674	3.978	3.827
	Standard Error	0.084	0.057	0.095	0.148	0.121	0.090	0.093	0.090	0.080	0.100	0.788	0.069
	95% CI	3.294–3.625	4.193–4.419	3.931–4.301	2.565–3.145	3.575–4.049	4.108–4.461	4.177–4.543	3.643–3.994	4.007–4.321	3.478–3.870	3.824–4.133	3.691–3.963
	Median	3.964	4.515	4.309	3.301	4.401	4.526	4.589	4.265	4.425	4.274	4.356	4.330

MAC, plain MacConkey agar; MACCEF, 3GC, third generation cephalosporin; MacConkey agar with 4 μg/mL of ceftriaxone

**Table 3:** Descriptive data on the distribution of *E*. *coli* growth across treatment and sampling day.

Growth Metric	Treatment	Descriptive Statistics	Sample Day				
			0	6	16	28	56
Log_10_ Total *E*. *coli* (MAC)	Treated	Sample Size	123	121	118	120	113
		Mean	4.701	3.000	4.388	4.758	4.903
		Standard Error	0.113	0.190	0.145	0.115	0.115
		95% CI	4.481–4.922	2.627–3.374	4.103–4.673	4.533–4.983	4.678–5.128
		Median	4.757	3.681	4.663	4.864	5.105
	Untreated	Sample Size	123	118	121	117	117
		Mean	4.581	4.876	4.776	4.761	4.775
		Standard Error	0.111	0.108	0.105	0.106	0.098
		95% CI	4.364–4.799	4.665–5.088	4.570–4.982	4.552–4.969	4.583–4.967
		Median	4.662	4.870	5.000	4.723	4.869
Log_10_ 3GC Resistant *E*. *coli* (MACCEF)	Treated	Sample Size	123	121	118	120	113
		Mean	0.635	1.564	1.095	0.554	0.554
		Standard Error	0.121	0.188	0.168	0.117	0.124
		95% CI	0.398–0.873	1.195–1.933	0.766–1.424	0.325–0.784	0.311–0.798
		75^th^ Percentile	0.000	3.556	2.603	0.000	0.000
	Untreated	Sample Size	123	118	121	117	117
		Mean	0.410	0.567	0.458	0.393	0.404
		Standard Error	0.097	0.121	0.111	0.102	0.107
		95% CI	0.219–0.600	0.331–0.804	0.241–0.675	0.193–0.592	0.194–0.615
		75^th^ Percentile	0.000	0.000	0.000	0.000	0.000
Log_10_ Growth Arithmetic Difference (Total– 3GC)	Treated	Sample Size	123	121	118	120	113
		Mean	4.066	1.436	3.292	4.204	4.349
		Standard Error	0.138	0.183	0.188	0.137	0.148
		95% CI	3.795–4.337	1.077–1.796	2.924–3.662	3.934–4.473	4.059–4.639
		Median	4.292	0.176	3.623	4.511	4.5798
	Untreated	Sample Size	123	118	121	117	117
		Mean	4.172	4.309	4.318	4.368	4.371
		Standard Error	0.133	0.138	0.138	0.116	0.115
		95% CI	3.911–4.432	4.038–4.580	4.047–4.590	4.141–4.595	4.144–4.597
		Median	4.326	4.538	4.722	4.526	4.593

MAC, plain MacConkey agar; 3GC, third generation cephalosporin; MACCEF, MacConkey agar with 4 μg/mL of ceftriaxone.

**Fig 2:**
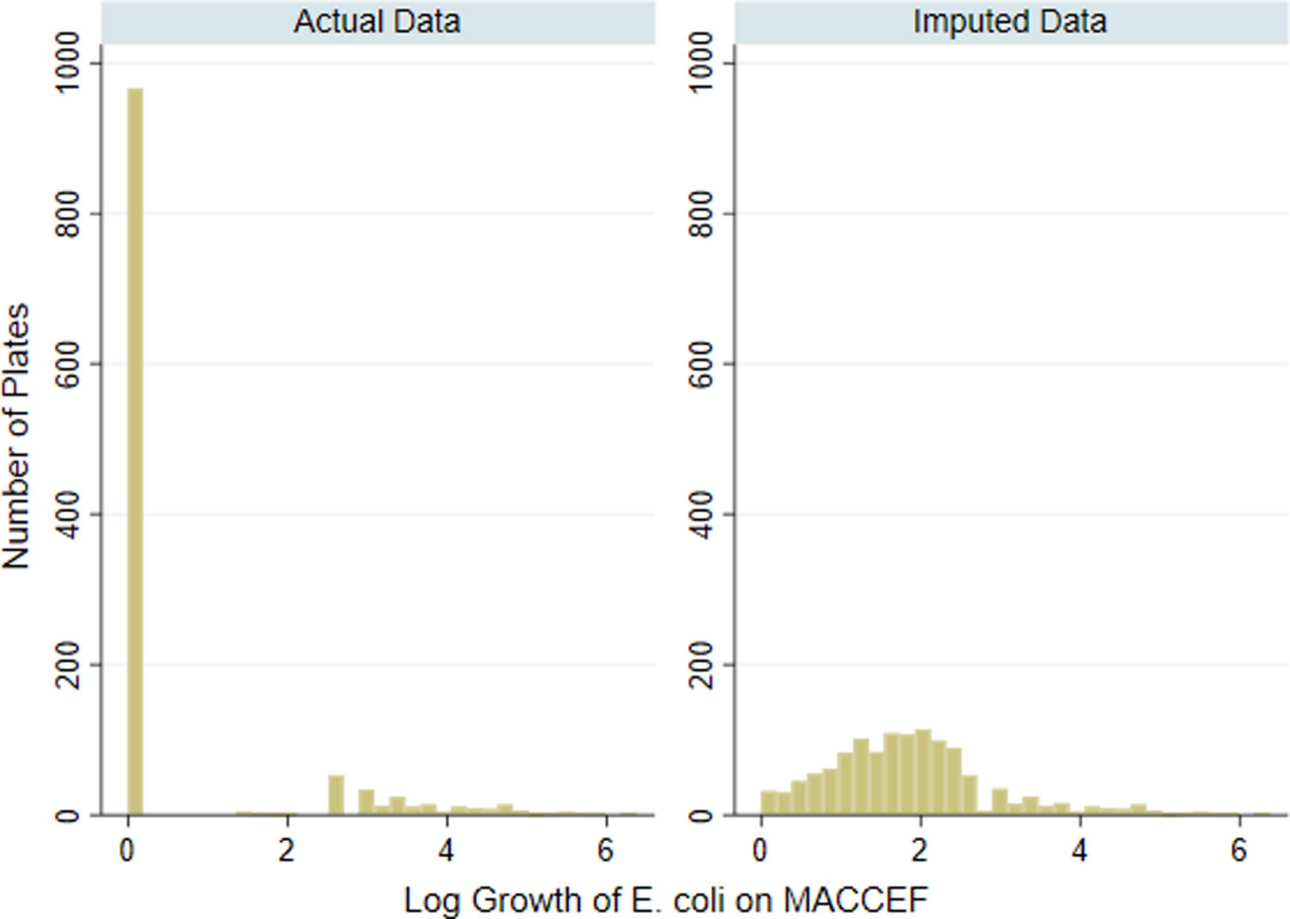
Visual representation of actual versus imputed third-generation cephalosporin resistant *E*. *coli* growth. a) Actual log_10_ CFU *E*. *coli* growth on MacConkey agar prepared with ceftriaxone (4μg/mL), b) Example of imputed log_10_ CFU *E*. *coli* growth on MacConkey agar prepared with ceftriaxone at 4μg/mL using multiple imputation procedures for values estimated below the limit of quantification.

Samples with quantifiable 3GC resistant *E*. *coli* growth were most prevalent on day 6 of the study (Table 4). Dairy Farm 3 had the largest number of samples with 3GC resistant *E*. *coli* both in total and on each sample day (Table 4). The distribution of CFU on MACCEF reflected a large number (n = 966) of plates with no detectable growth (Fig 2A). It is unlikely these counts were truly zero. Because of this zero-inflation, modeling the count data using linear regression was not ideal due to inappropriate residuals, non-normal error distribution, heightened instability, and enlarged coefficients. By utilizing multiple imputed data of 3GC growth below the limits of detection, model estimates increased in stability due to the more normalized data distribution (Fig 2B). Of the isolates presumed to be *E*. *coli* and selected for further testing (n = 1603), 100 percent tested as indole positive and 98.6% of these were later confirmed via MALDI—TOF MS as *E*. *coli* providing confidence in our ability to phenotypically identify and include only *E*. *coli* in the colony counting procedure.

**Table 4:** Distribution of samples testing positive for detectable levels of 3GC resistant *E*. *coli* growth by sample day and dairy farm.

	Dairy Number			
Growth on MacConkey Agar with Ceftriaxone (4 μg/mL)	Dairy Farm 1	Dairy Farm 2	Dairy Farm 3	Total
Sample Day 0				
Growth	4 (4.88%)	9 (10.71%)	29 (36.25%)	42 (9.76%)
No Growth	78 (95.12%)	75 (89.29%)	51 (63.75%)	204 (90.24%)
Total	82	84	80	246
Sample Day 6				
Growth	18 (21.43%)	8 (10.53%)	41 (51.90%)	67 (28.03%)
No Growth	66 (78.57%)	68 (89.47%)	38 (48.10%)	172 (71.97%)
Total	84	76	79	239
Sample Day 16				
Growth	9 (10.84%)	6 (7.79%)	35 (44.30%)	50 (20.92%)
No Growth	74 (89.16%)	71 (92.21%)	44 (55.70%)	189 (79.08%)
Total	83	77	79	239
Sample Day 28				
Growth	11 (13.41%)	6 (7.89%)	17 (21.52%)	34 (14.35%)
No Growth	71 (86.59%)	70 (92.11%)	62 (78.48%)	203 (85.65%)
Total	82	76	79	237
Sample Day 56				
Growth	10 (12.99%)	1 (1.33%)	21 (26.92%)	32 (13.91%)
No Growth	67 (87.01%)	74 (98.67%)	57 (73.08%)	198 (86.09%)
Total	77	75	78	230

### Mixed model of total *E*. *coli* counts

**Fig 3:**
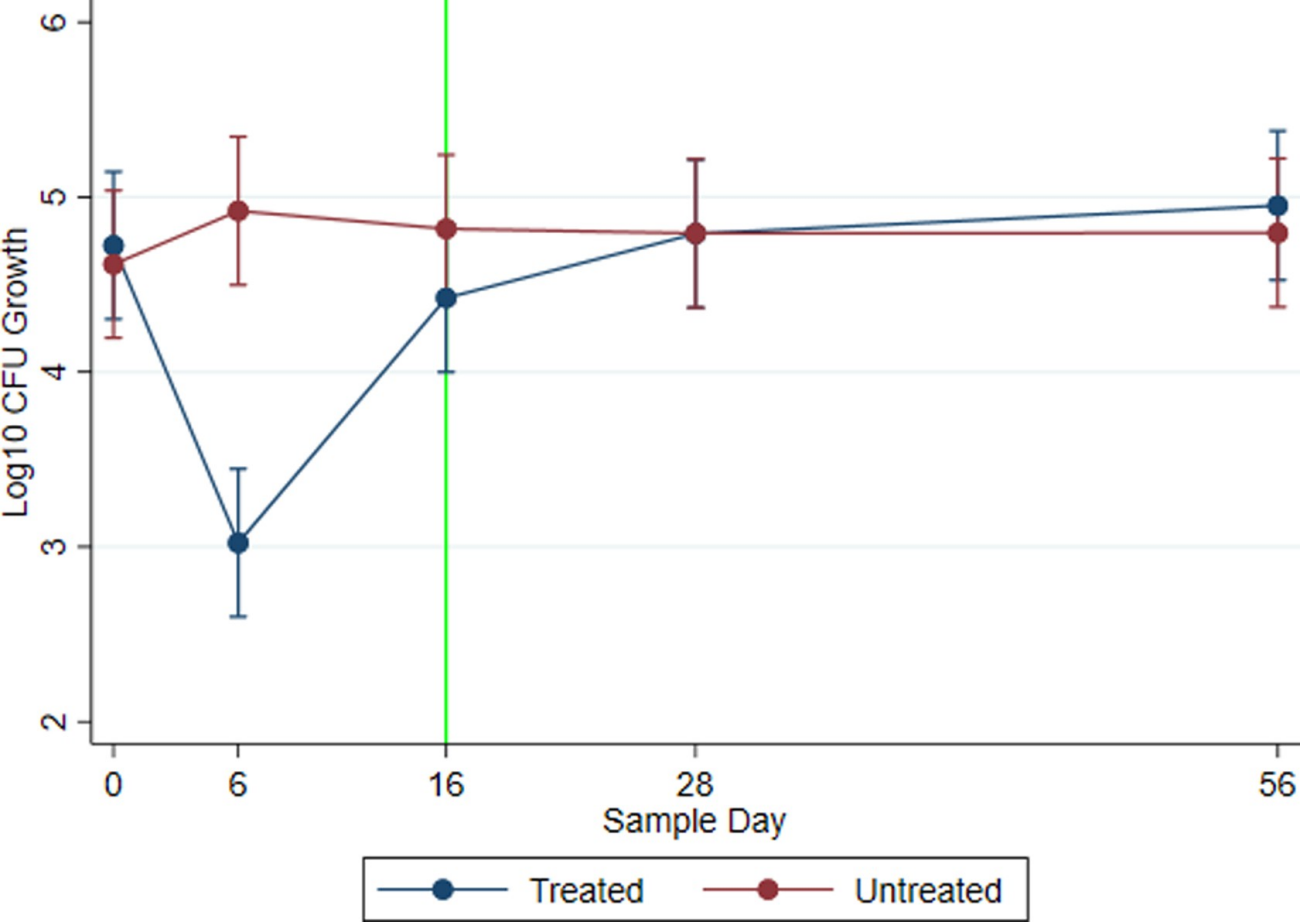
Total log_10_ *E*. *coli* growth. Total log_10_ CFU *E*. *coli* growth on plain MacConkey agar plotted across sample day by treatment group. Data points represent modeled marginal mean estimates and 95 percent confidence intervals. CCFA-treated cows with metritis are represented by the blue line, while untreated pair-matched controls are represented by the red line. The vertical green line represents the first day that a treated cow is eligible (per drug label) to be shipped for slaughter.

Mixed-effect linear regression was utilized to evaluate *E*. *coli* counts on a log_10_ scale across sampling days. Fig 3 represents modeled marginal mean *E*. *coli* log_10_ CFU estimates with 95% confidence intervals on plain MAC agar by treatment group and across study days for all three farms. Both treated and untreated groups had similar quantities of enteric *E*. *coli* pre-treatment (Day 0: P = 0.927; Treated: log_10_ CFU: 4.723; 95% CI: 4.301–5.145; Untreated: log_10_ CFU: 4.616; 95% CI: 4.194–5.037). However, following two sequential doses of CCFA, the modeled count of *E*. *coli* in the treated group decreased by nearly two log_10_ CFU below that of the untreated group (*P* < 0.0001: Treated 95% CI: 2.600–3.445; Untreated 95% CI: 4.497–5.345). At day 16, the first eligible date for slaughter after a two-dose treatment regimen with CCFA, the *E*. *coli* population of the treated group rebounded but remained somewhat different (*P* = 0.053; Treated 95% CI: 3.999–4.847; Untreated 95% CI: 4.396–5.242) from the control group. By study days 28 and 56, the *E*. *coli* populations of the treated group showed levels similar to that of the untreated group. Historical usage of ceftiofur formulations did not result in significantly different total *E*. *coli* growth counts with Dairy Farm 2 as referent category (Dairy Farm 1: *P* = 0.854, 95% CI = -0.685–0.828; Dairy Farm 3: *P* = 0.144, 95% CI = -0.192–1.321). Random effects attributed to dairy farm accounted for 11.4 percent of the variance components in the model with 9.7 percent attributed to animal.

### Mixed model of 3GC resistant *E*. *coli* counts

**Fig 4:**
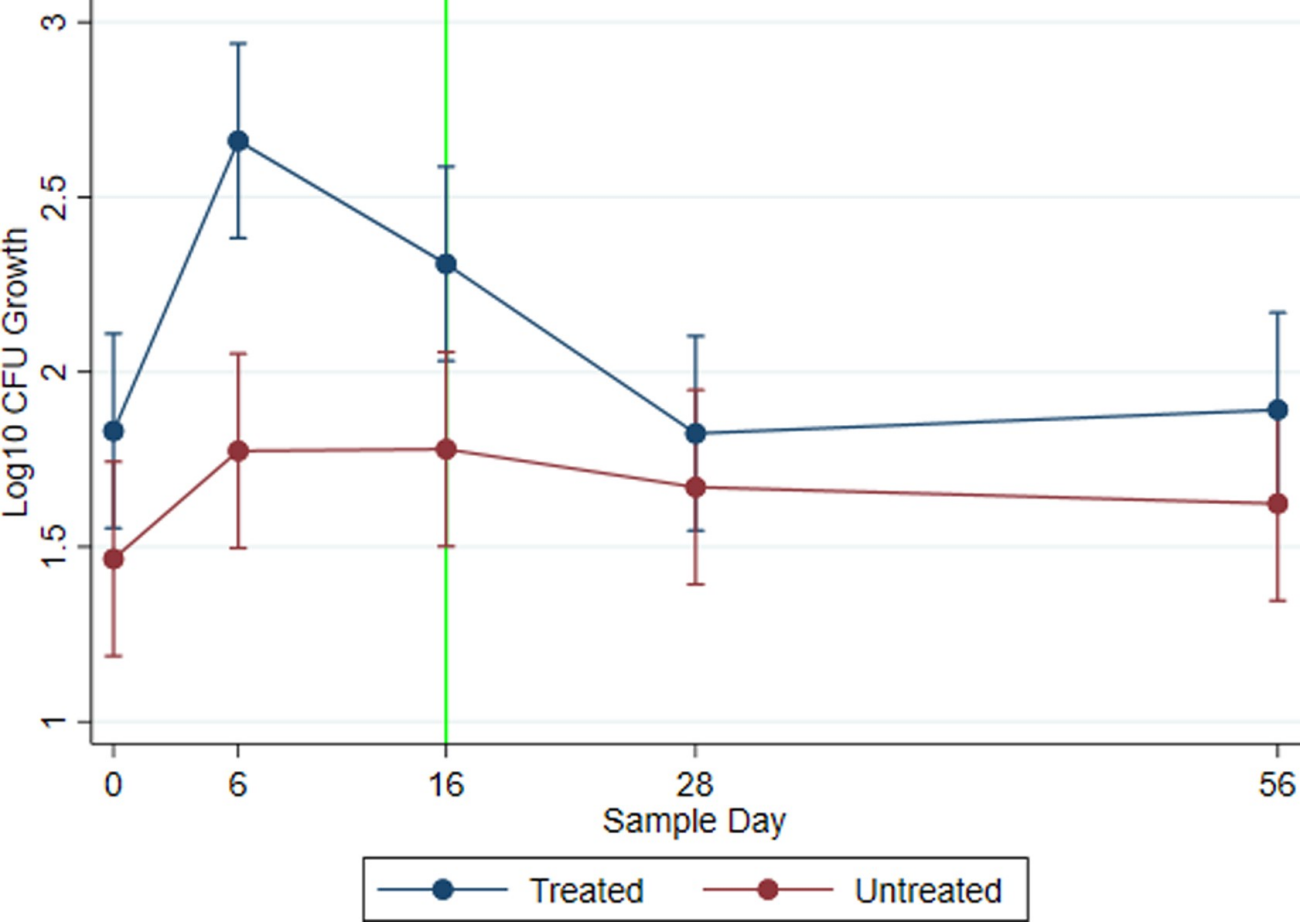
Third-generation cephalosporin resistant *E*. *coli* growth. Total log_10_ CFU *E*. *coli* growth on MacConkey agar prepared with ceftriaxone (4 μg/mL) plotted across sample day and by treatment group for all three dairy farms. Data points represent modeled marginal mean estimates with 95% confidence intervals. Zero counts were imputed across levels for data points below the limit quantification (see Fig 2A and 2B). CCFA-treated cows with metritis are represented by the blue line, while untreated pair-matched controls are represented by the red line. The vertical green line represents the first day that a treated cow is eligible (per drug label) to be shipped for slaughter.

Fig 4 depicts the quantity of enteric *E*. *coli* resistant to ceftriaxone at an MIC ≥ 4 μg/mL by treatment and across study day modeled by mixed effects linear regression. Following the two-dose treatment with CCFA, the 3GC resistant *E*. *coli* population of the treated group increased to 1.5 log_10_ CFU above the untreated group by study day 6 (*P* < 0.0001; Treated 95% CI: 2.383–2.938; Untreated 95% CI: 1.496–2.052). On study day 16, the population of 3GC resistant bacteria in the treatment group had been reduced by 0.5 log_10_ CFU, remaining higher, though not significantly different, than the untreated group (*P* = 0.134; Treated 95% CI: 2.031–2.587; Untreated 95% CI: 1.501–2.057). As time progressed to days 28 and 56, the population of 3GC resistant *E*. *coli* further decreased towards pre-treatment levels. Cows with a lactation number greater than three had higher relative quantities of 3GC resistant *E*. *coli* than first lactation animals (*P* = 0.002; 95% CI: log_10_ CFU difference of 0.114–0.501). Historical ceftiofur usage with Dairy Farm 2 as referent did not yield a statistically significant difference regarding 3GC resistant *E*. *coli* growth when compared to Dairy Farm 1 (*P* = 0.341, 95% CI: -0.199–0.576), but was substantively different when compared to Dairy Farm 3 (*P* < 0.0001, 95% CI: 0.645–1.405). Following imputation and incorporating all fixed and random effects, 7.63 percent of the variance in the model was attributed to the dairy farm with 9.8 percent attributed to animal.

### Mixed model of difference between total and 3GC resistant *E*. *coli* counts

**Fig 5:**
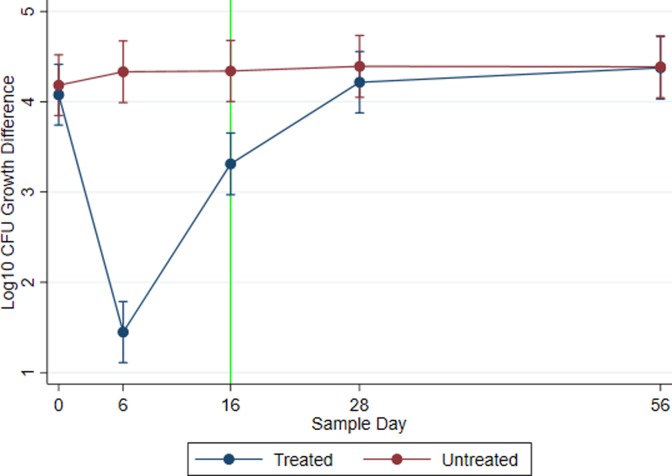
Total versus third-generation cephalosporin resistant *E*. *coli* growth differences. Overall (across three dairy farms) log_10_ marginal predicted mean arithmetic differences of *E*. *coli* CFU growth on plain MacConkey agar versus the same agar with ceftriaxone (4 μg/mL). Smaller differences indicate a higher proportion of total *E*. *coli* harbor resistance to third-generation cephalosporins. Line graphs are plotted across sample days and by treatment group. Each plotted point indicates a corresponding modeled marginal mean and 95 percent confidence interval. Log_10_ CFU *E*. *coli* growth differences for CCFA treated cows with metritis are illustrated by blue lines; whereas, red lines represent pair-matched untreated cows. The vertical green line represents the first day that a treated cow is eligible (per drug label) to be shipped for slaughter.

The outcome modeled as the arithmetic difference between the total enteric *E*. *coli* (MAC) and the 3GC resistant enteric *E*. *coli* (MACCEF) CFU by treatment across study days using mixed effects linear regression is displayed in Fig 5. This analysis utilized the original MACCEF data, not imputed values. The rationale for this analysis was that as the baseline levels of total coliforms change, the observed changes in quantity of resistance resulted from shifts in either the numerator, denominator, or else both. The difference in the relative quantities across sampling days for the untreated group remained constant over time (day 0 95% CI: 3.847–4.520; day 6 95% CI: 3.990–4.673; day 16 95% CI: 4.002–4.679; day 28 95% CI: 4.050–4.735; day 56 95% CI: 4.044–4.729). At baseline day 0, the arithmetic difference in the relative quantities was at similar levels for both treated and untreated groups (*P* = 0.775; Treated: log_10_ CFU: 4.080; 95% CI: 3.740–4.420; Untreated: log_10_ CFU: 4.185; 95% CI: 3.846–4.525). However, following treatment with CCFA, the log_10_ arithmetic difference between total *E*. *coli* and 3GC resistant *E*. *coli* CFU was reduced to 1.5 log_10_; meaning, of bacteria remaining after treatment, approximately one in 32 total colony-forming units were resistant to ceftriaxone. Of note, this differed significantly from the untreated group (*P* < 0.0001; Treated 95% CI: 1.111–1.788; Untreated 95% CI: 3.990–4.673). This difference was also observed on the first-eligible slaughter date (day 16) (*P* = 0.001; Treated arithmetic differences in log_10_ CFU 95% CI: 2.970–3.653; Untreated 95% CI: 4.002–4.680). On study day 16, approximately one in 1,250 *E*. *coli* CFU were 3GC resistant. By study days 28 and 56, the difference in total and resistant *E*. *coli* populations in the treatment group was not significantly different (P > 0.05) from levels from the untreated group (Day 28: *P* = 0.808; Treated 95% CI: 3.877–4.556; Untreated 95% CI: 4.050–4.735; Day 56: *P* = 0.775; Treated 95% CI: 4.029–4.722; Untreated 95% CI: 4.044–4.729). Similar to 3GC resistant *E*. *coli* models, historical ceftiofur usage with Dairy Farm 2 as referent did not yield significantly different values from Dairy Farm 1 (*P* = 0.135, 95% CI: -0.638–0.086), but did differ statistically from Dairy Farm 3 (*P* = 0.009, 95% CI: -0.848 –-0.122). In this analysis, dairy farm accounted for 2.3 percent of the total variance and animal accounted for 6.2 percent.

**Fig 6:**
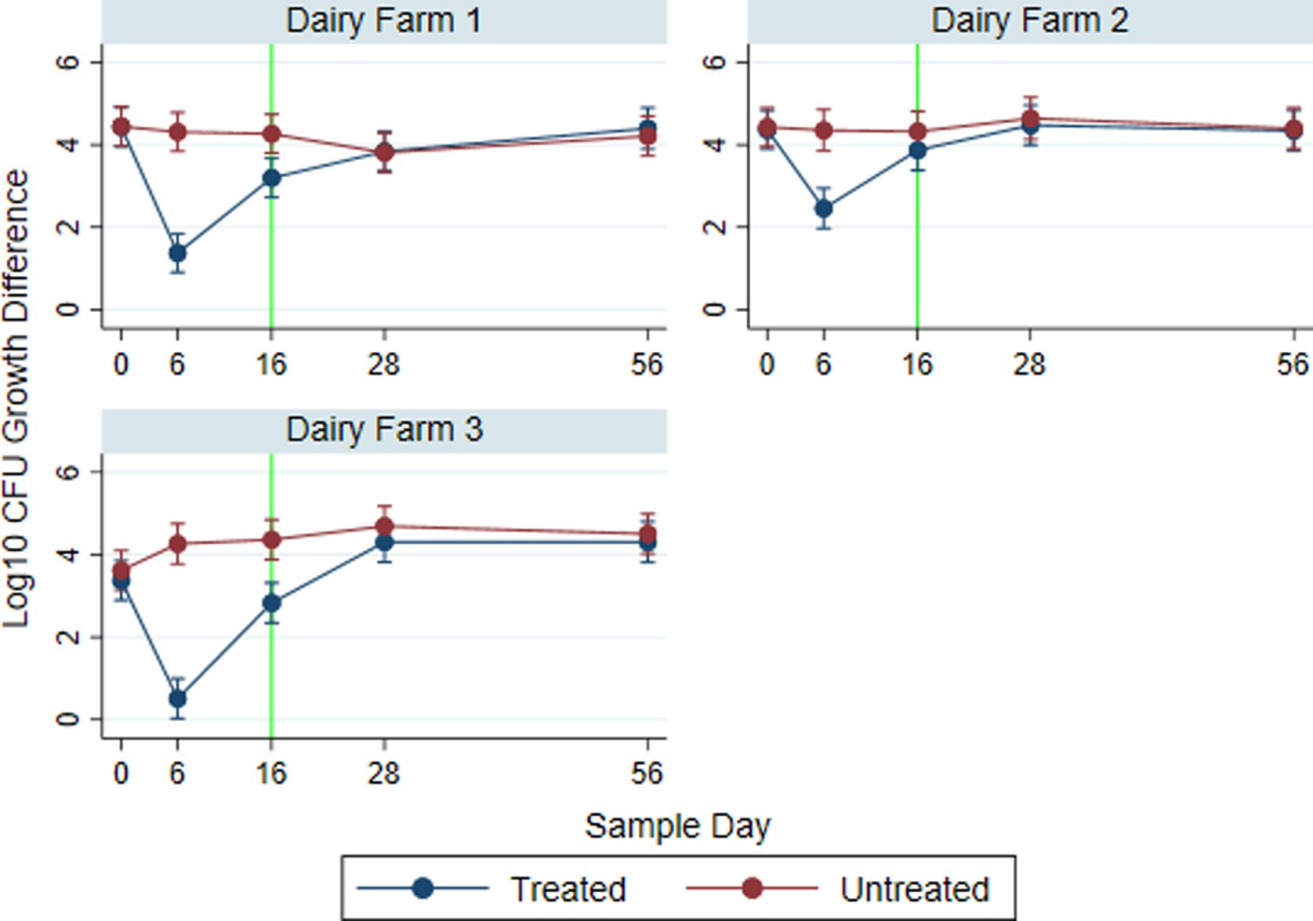
Total and third-generation cephalosporin resistant *E*. *coli* growth difference by dairy farm. Log_10_ marginal predicted mean arithmetic differences of *E*. *coli* CFU growth on plain MacConkey agar versus the same agar with ceftriaxone (4 μg/mL). Smaller differences indicate a higher proportion of total *E*. *coli* harbor resistance to third-generation cephalosporins. Line graphs (one subgraph for each dairy farm) are plotted across sample days and by treatment group. Each plotted point indicates a corresponding modeled marginal mean and 95 percent confidence interval. Log_10_ CFU *E*. *coli* growth differences for CCFA treated cows with metritis are illustrated by blue lines whereas red lines represent pair-matched untreated cows. The vertical green line represents the first day that a treated cow is eligible (per drug label) to be shipped for slaughter.

Due to the variability resulting from dairy farm, mixed effect linear regression with three-way interaction of farm, treatment, and sample day was performed on the arithmetic difference in *E*. *coli* growth on MAC and MACCEF agars. Fig 6 shows the breakdown of the difference of total and resistant *E*. *coli* populations by dairy farm. The difference in counts for untreated groups remained constant throughout. However, on both study days 6 and 16, the Dairy Farm 3 treatment group exhibited the smallest difference between total and resistant *E*. *coli* populations, followed by Dairy Farm 1 and Dairy Farm 2. Fig 6 shows the starting log_10_ CFU arithmetic differences between total and 3GC resistant populations are a single log_10_ CFU lower on Dairy Farm 3 than those same paired differences on Dairy Farms 1 and 2; meaning, dairy 3 has a higher proportional level of resistant *E*. *coli* than the other two locations. The ratio of 3GC resistant to total *E*. *coli* increases significantly in the treated group compared to the untreated group on day 6 on all three farms: Dairy Farm 1 (*P* < 0.0001; Treated 95% CI: 0.912–1.846; Untreated 95% CI: 3.851–4.785), Dairy Farm 2 (*P* < 0.0001; Treated 95% CI: 1.976–2.945; Untreated 95% CI: 3.859–4.853), and Dairy Farm 3 (*P* < 0.0001; Treated 95% CI: 0.034–0.991; Untreated 95% CI: 3.785–4.753). These differences begin to decrease as time progresses. However, the level of significance concerning these differences on day 16 (slaughter eligibility) varies greatly by farm. The differences observed on day 16 for the treated groups are significantly different from the untreated group on Dairy Farm 1 (*P* = 0.027; Treated 95% CI: 2.728–3.673 Untreated 95% CI: 3.807–4.740) and 3 (*P* = 0.008; Treated 95% CI: 2.352–3.321; Untreated 95% CI: 3.892–4.848), but are non-significantly different on Dairy Farm 2 (*P* = 0.081; Treated 95% CI: 3.385–4.366; Untreated 95% CI: 3.844–4.812). Because Dairy Farm was included in this model as a fixed effect, unlike overall models where it was included as a random effect, historical ceftiofur usage was not considered due to its collinearity with farm.

## Discussion

The results of this study highlight the impact of a two-dose treatment of CCFA for metritis on the dynamics of total and 3GC resistant *E*. *coli* populations over time. The data show that a 13-day withholding period from the final administration of a two-dose treatment of CCFA for metritis may be insufficient to ensure that 3GC resistant *E*. *coli* populations return to baseline levels in all treated cattle and mitigate the risk of these organisms “escaping from the farm” as illustrated in the risk assessment of Hurd [28, 29]. It has previously been reported that at the aggregate level, dairy farms employing ceftiofur are 25 times more likely to have detectable *E*. *coli* with reduced susceptibility to ceftriaxone [14]. The fact that all three dairy farms in our study harbored 3GC resistant *E*. *coli* is consistent with earlier work that found 92 percent of tested herds had ceftriaxone-resistant *E*. *coli* and 88 percent of tested herds used ceftiofur [26]. While the number of samples containing resistant *E*. *coli* varied, each dairy farm had detectable *E*. *coli* with phenotypic resistance to ceftriaxone. As expected, based on work by Lowrance et al. [24], Kanwar et al. [34] and Singer et al. [35], the total *E*. *coli* count in cattle decreased significantly following treatment with CCFA, but rebounded over time.

### 3GC-resistant *E*. *coli*

Furthermore, 3GC resistant *E*. *coli* populations were significantly higher in treated groups than untreated groups on day 6. This was observed even with large numbers of zero counts and remained after the large zero counts were imputed to distribute those values across levels below detection. It seemed implausible that the distribution contained true zero values below the limit of quantification for the assay (i.e., that the zero values were truly zero). A number of techniques have been deployed and evaluated to deal with this problem [34]. While not significant (*P*<0.05) overall, with robust marginal mean confidence intervals, treated animals harbored higher levels of 3GC resistant *E*. *coli* than untreated groups on study day 16. While there were a few samples with 3GC resistance among control animals (75^th^ percentile = 0), there remained elevated levels in treated cows (75^th^ percentile = 2.603 log_10_ CFU). It has been suggested such increases in total 3GC resistant *E*. *coli* counts may be due to an increased ability to detect them due to the suppression of total growth [36]; however, at day 16 total *E*. *coli* CFU (MAC) did not differ significantly between groups. The probability of 3GC resistant *E*. *coli* increased in cows with parities above three. This may be a result of these animals having been repeatedly treated with or had exposure to CCFA throughout their life.

### Arithmetic difference between total and 3GC-resistant *E*. *coli* CFU

When evaluating the arithmetic difference in total and 3GC resistant *E*. *coli* growth, higher proportions of colonies were resistant to 3GCs in the treated groups on days 6 and 16 than among untreated groups. However, the level of significance regarding these counts varied by dairy farm. This may be a result of increased 3GC resistant *E*. *coli* populations in environmental manure accumulating based on higher levels of historical ceftiofur usage. Due to the significance of historical usage in impacting levels of 3GC resistant *E*. *coli*, we hypothesize such historical dairy features play a role in expanding 3GC populations in the environment. This helps sustain a higher proportion of 3GC resistant *E*. *coli* in the gut of untreated animals, and leads to an increased time for resistant bacterial populations to return to pre-treatment levels following a two-dose CCFA treatment for metritis. However, due to our small environmental sampling size, limited number of farms, and historical usage data consisting of only the year prior to the start of the study, further work is required to properly evaluate the role of historical usage.

It is also important to note the variation of systemic ceftiofur usage. A quarter of the historical usage on Dairy Farm 2 was intramammary, which would be less likely to affect levels of 3GC resistance among fecal *E*. *coli*. On the other hand, Dairy Farm 1 did not use any intramammary ceftiofur and a smaller portion of ceftiofur use on Dairy Farm 3 was intramammary which helps explain their elevated 3GC resistance among fecal bacteria. The currently observed differences in ceftiofur administration across multiple farms may be a result of other variables not considered for the purpose of our study, such as the incidence of foot rot and bovine respiratory disease, or the severity or form of metritis at the time of diagnosis. Additionally, since rates of treatment were collected from farm records and therefore were farm-level reported, actual treatment rates may differ from calculated values. Unfortunately, we were unable to aggregate historical usage data for additional antibiotics used on the farms in the previous year. One might imagine farms using ceftiofur at elevated rates may be using greater amounts of other antibiotics, but this is far from certain and an important area to consider in future research. Across all dairy farms, the proportion of total and 3GC resistant populations return to pre-treatment levels by days 28 and 56. It has been hypothesized that bacteria with 3GC resistance are less able to compete with general *E*. *coli* populations after the removal of the antibiotic selection pressure due to the fitness cost of harboring a functional AmpC *bla*_CMY-2_ or another ESBL gene [24, 37], thereby explaining their decline post-treatment.

### Recommendations

These results suggest a 13-day withholding period following CCFA treatment does not universally provide adequate time to allow for 3GC resistant populations of enteric *E*. *coli* to return to baseline levels. While these changes in 3GC resistant *E*. *coli* populations may seem inconsequential when evaluating a gram of feces, they are not when considering the many kg of fecal matter produced by the animal. There are well-documented instances of beef being contaminated with AMR Enterobacteriaceae, based on meat samples taken in butcher shops [36] and grocery stores [21–23] and with potential transmission from such sources to the human population [30]. We hypothesize, based upon our findings, there may be an increased risk posed to public health should an animal be culled at the current 13-day withholding period due to elevated levels of 3GC resistant fecal *E*. *coli*. Studies determining slaughter withholding periods tend to be performed in healthy animals [38]; however, the pharmacokinetics and pharmacodynamics are likely to vary between healthy and clinically ill populations [39] due to pathophysiological changes associated with illness. Our study provides insight into the fluctuation of bacterial populations in ill animals undergoing treatment and their healthy counterparts. While *E*. *coli* were not typed as commensal or pathogenic, because *E*. *coli* is an indicator species for gram-negative bacteria, these findings may model patterns that could be observed in other enteric gram-negative pathogens such as *Salmonella* spp. [24, 36].

Further studies should be performed to evaluate the effects of such treatment on 3GC resistance and shedding of *Salmonella*, along with quantitative polymerase chain reactions (qPCR) or shotgun metagenomics to evaluate the absolute and relative change in resistance genes found within samples across time. Based on these data, an additional withholding period prior to slaughter would be advisable on farms with a long-standing history of cephalosporin use, or with known moderate to high levels of 3GC resistance, to reduce the risk to public health and improve antimicrobial stewardship. While a decade ago, Daniels et al. reported the frequency with which ceftiofur is administered does not impact levels of commensal *E*. *coli* containing the *bla*_CMY-2_ gene at the herd-level [35]; discrepancies likely exist regarding genotypic and phenotypic AMR indicators based upon the level of antibiotic usage on dairy farm [40]. Bacteria from dairy farms with low levels of antibiotic usage may still harbor bacteria with genetic elements encoding for resistance, but not at high enough levels to detect phenotypic resistance; meanwhile, the opposite may be observed on farms with high levels of antibiotic usage [40].

### Study limitations

Limitations of this study include its small geographic region. Our study included only dairy farms from western Texas and eastern New Mexico, which tend to have warmer, drier climates, versus other dairy production regions, such as Wisconsin and New York. Differences in climate may impact bacterial survival outside the host, affecting the oral-fecal transmission dynamics within the farm, bacterial fitness in the environment, and the ability of such bacteria to further distribute resistance genes in the environment. In addition to climate, environment and resource availability could impact 3GC resistant bacterial populations or else herd management techniques resulting from those factors [41]. Studies across diverse regions of the U.S. with high levels of dairy production would aid in providing additional insight into the generalizability of our findings. It is beneficial to sample animals from a dairy farm multiple times to evaluate temporal dynamics; however, due to the resources required for repeated intensive temporal sampling only three dairy farms could be enrolled in the study. As a result, the generalizability of our data is restricted. Further, our use of multiple imputations to complete the truncated left tail of the empirical distribution and assist in meeting model assumptions may have introduced bias into the models.

## Conclusions

The findings of this study suggest that the currently labeled 13-day slaughter-withholding period following a second dose of CCFA is generally inadequate to allow levels of 3GC resistant bacteria to return to baseline levels in treated dairy cows housed on dairy farms with elevated levels of resistance to third-generation cephalosporins. Existing slaughter withholding times designed to avoid pharmaceutical residues in meat products provide no microbial safety assurances once bacterial resistance establishes in agricultural environments. A longer stakeholder-initiated voluntary slaughter-withholding period could be deployed to reduce the risk of fecal contamination at slaughter with antimicrobial resistant enteric bacteria. Dairy-specific stewardship approaches targeting practices with higher risk of selecting for antimicrobial resistance would be beneficial in mitigating resistance. Further research into the genetic diversity of resistance will provide more information into the mechanisms at play and the added potential for 3GC resistance co-selection via alternative antimicrobial classes that may be employed instead of cephalosporins.

## Supporting information

S1 Dataset Animal data. (XLSX)

S2 Dataset Environmental data. (XLSX)
